# Supplementary material for: Predicting electronic screening for fast Koopmans spectral functional calculations
Source: NPJ Comput Mater. 2024 Dec 20;10(1):299. doi: 10.1038/s41524-024-01484-3 (PMC11659161; doi:10.1038/s41524-024-01484-3)
Supplement: Supplementary file 1 — Supplementary information [file 41524_2024_1484_MOESM1_ESM.pdf]

# Supplementary information: Predicting electronic screening for fast Koopmans spectral functional calculations

Yannick Schubert[1], Sandra Luber[1], Nicola Marzari[2,3], Edward Linscott[3,4*]

[1]Department of Chemistry, University of Zurich, 8057 Zurich, Switzerland.
[2]Theory and Simulations of Materials (THEOS) and National Centre for Computational Design and Discovery of Novel Materials (MARVEL), École Polytechnique Fédérale de Lausanne, 1015 Lausanne, Switzerland.
[3]Center for Scientific Computing, Theory and Data, Paul Scherrer Institute, 5352 Villigen PSI, Switzerland.
[4]National Centre for Computational Design and Discovery of Novel Materials (MARVEL), Paul Scherrer Institute, 5352 Villigen PSI, Switzerland.

*Corresponding author(s). E-mail(s): edward.linscott@psi.ch;

## Supplementary Discussion 1: Correlation between self-Hartree energies and screening parameters

### Acenes

Correlations exist between the self-Hartree energies and screening parameters for different acenes (benzene, naphthalene, and anthracene), as shown in Supplementary Figure 1. In contrast to the liquid water and perovskite studied in the main text, these are non-periodic systems and correspondingly instead of Wannier orbitals the variational orbitals are chosen as either Kohn-Sham or Perdew-Zunger orbitals. The former will be delocalized across the molecule; the latter will be more localized. The correlations are stronger in the case of the Kohn-Sham orbitals.

### Water

Testing the same correlation for the water system, we find that the correlation is much weaker (see Supplementary Figure 2). For water, the empty states and the occupied states have very similar screening parameters but very different self-Hartree energies. This suggests that the self-Hartree energies alone don't contain sufficient information to predict the screening parameters. If we had predicted occupied and empty states with the same sH model instead of two separate ones, we would have obtained similarly poor results as for the mean model.

### $CsSnI_3$

For $CsSnI_3$, there is almost no correlation between the self-Hartree energies and the screening parameters of MLWFs (see Supplementary Figure 2). In the individual clusters of data, there are many orbitals with very similar self-Hartree energies but different screening parameters. Moreover, the clusters as a whole seem to show little correlation with the self-Hartree energies.

## Supplementary Discussion 2: Basis functions details

For the basis functions used to represent orbital densities, we adapt the choice (following Ref. 1 and many others) of taking real-valued spherical harmonics as angular basis functions and Gaussian basis functions as radial basis functions.

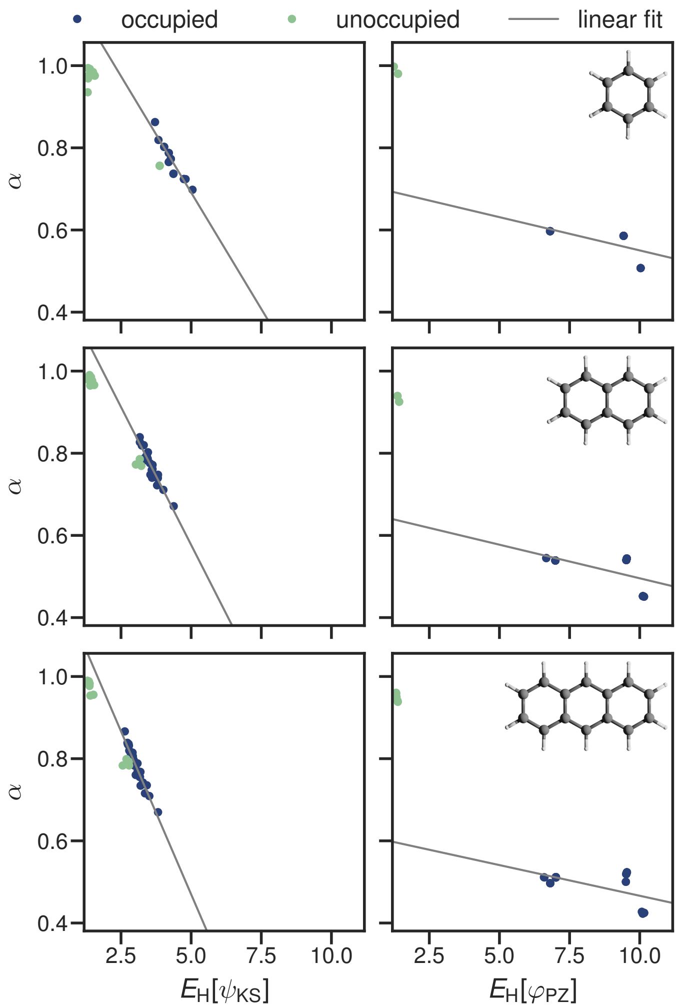


**Supplementary Figure 1**: **Correlation between the self-Hartree energies and the screening parameters for three different acenes: benzene, naphthalene, and anthracene.** The left panel shows the results with Kohn-Sham initial orbitals and the right panel the results with Perdew-Zunger initial orbitals. Green dots correspond to filled and red dots to empty orbitals. The linear interpolation (black line) was performed using only the occupied orbitals.

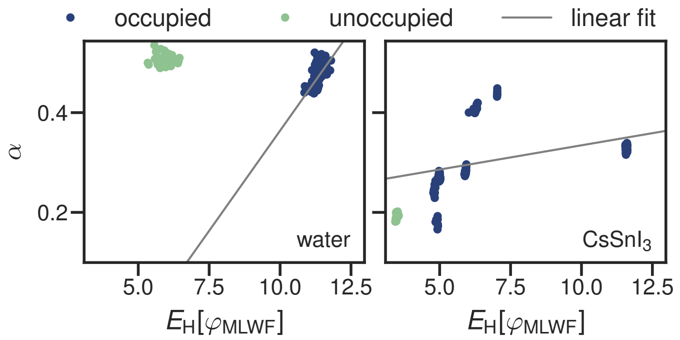


**Supplementary Figure 2**: **Correlation between the self-Hartree energies and the screening parameters of the MLWFs of water and $CsSnI_3$.** The line is fitted to the occupied orbital data.

## Angular basis functions

The real-valued spherical harmonics are given by

$$Y_{lm}(\theta,\varphi) = \begin{cases} \sqrt{2}(-1)^m \Im\left[Y_l^{|m|}(\theta,\varphi)\right] & \text{if } m < 0 \\ Y_l^0 & \text{if } m = 0 \\ \sqrt{2}(-1)^m \Re\left[Y_l^m(\theta,\varphi)\right] & \text{if } m > 0 \end{cases}$$

and define an orthogonal and complete set of angular basis functions. Here, $Y_l^m$ are the complex orthonormalized spherical harmonics:

$$Y_l^m(\theta, \varphi) = \sqrt{\frac{(2l+1)}{4\pi}\frac{(l-m)!}{(l+m)!}} P_l^m(\cos(\theta)) e^{im\varphi} \tag{1}$$

and $P_l^m$ are the associated Legendre polynomials. The expansion into spherical harmonics are truncated after some maximum value $l_{\mathrm{max}}$.

## Radial basis functions

For the radial basis functions, we construct a set of orthonormal basis functions

$$g_{nl}(r) = \sum_{n'=1}^{n_{\mathrm{max}}} \beta_{nn'l} \phi_{n'l}(r)$$

out of a set of linearly independent Gaussians:

$$\phi_{nl}(r) = r^l e^{-\gamma_{nl} r^2}.$$

The decay parameters $\gamma_{nl}$ are chosen such that $\phi_{nl}$ decays to a threshold value of $10^{-3}$ for cutoff radii taken on an evenly spaced grid from $r_{\mathrm{min}}$ to $r_{\mathrm{max}}$. This means for each $n \in \{1, \ldots, n_{\mathrm{max}}\}$ the cutoff radius is given by

$$r_{\mathrm{thr},n} = r_{\mathrm{min}} + \frac{n-1}{n_{\mathrm{max}}-1}(r_{\mathrm{max}} - r_{\mathrm{min}})$$

The coefficients $\beta_{nn'l}$ are obtained with a Löwdin orthogonalization procedure [2]: $\boldsymbol{\beta}_l = \mathbf{S}_l^{-1/2}$, where

$$(\boldsymbol{S}_l)_{nn'} = \langle \phi_{nl} | \phi_{n'l} \rangle = \int_0^\infty dr\, r^{2(l+1)} e^{-(\gamma_{nl}+\gamma_{n'l})r^2}, \tag{2}$$

## Reconstructed orbital densities

In Supplementary Figures 3 and 4 we demonstrate the efficacy of the orbital density descriptors by comparing variational orbital densities against reconstructed densities i.e. densities obtained by performing the decomposition into a truncated basis set as described above and in the main text, and then taking the linear combination of the basis functions with the obtained expansion coefficients. Without the truncation, the original and reconstructed densities should be identical.

## Supplementary Discussion 3: Sensitivity of the eigenvalues to the screening parameters

In the main text it was shown that the Koopmans correction shifts DFT Kohn-Sham eigenvalues by

$$\Delta\varepsilon_i = \varepsilon_i^{\mathrm{KI}} - \varepsilon_i^{\mathrm{DFT}} = \sum_{jk} \alpha_j U_{ij} U_{ki}^\dagger \langle \varphi_k | \hat{v}_j^{\mathrm{KI}} | \varphi_j \rangle \tag{3}$$

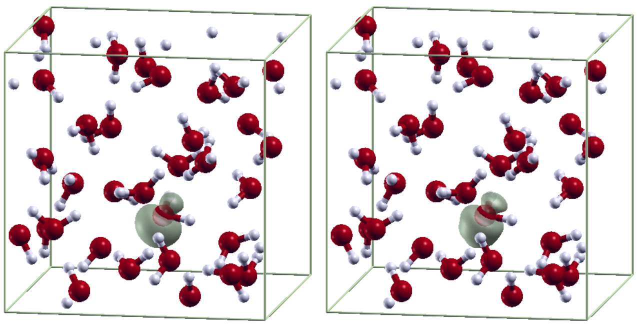

(a) original (b) reconstructed

**Supplementary Figure 3: Original and reconstructed occupied $sp^3$ Wannier function centered on an oxygen atom in the water system.** The isosurfaces are plotted at $0.005 a_0^{-3}$.

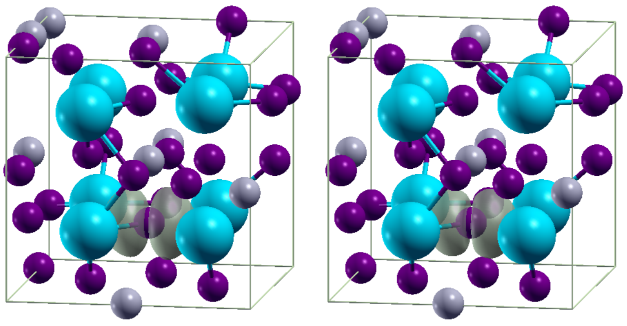

(a) original (b) reconstructed

**Supplementary Figure 4**: **Original and reconstructed occupied** $p$ **Wannier orbital centered on an iodine atom in** $\mathbf{CsSnI_3}$**.** The isosurfaces are plotted at $0.0005a_0^{-3}$.

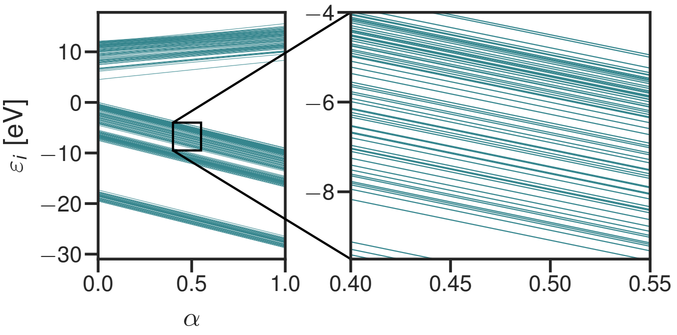


**Supplementary Figure 5**: **The KI@PBE eigenvalues of a liquid water system as a function of the screening parameter.** The same screening parameter was used for every variational orbital in the system. The eigenvalues energies are provided relative to the HOMO energy of the DFT solution. The inset shows the quasi-particle energies of the uppermost occupied states across the range of values measured *ab initio*.

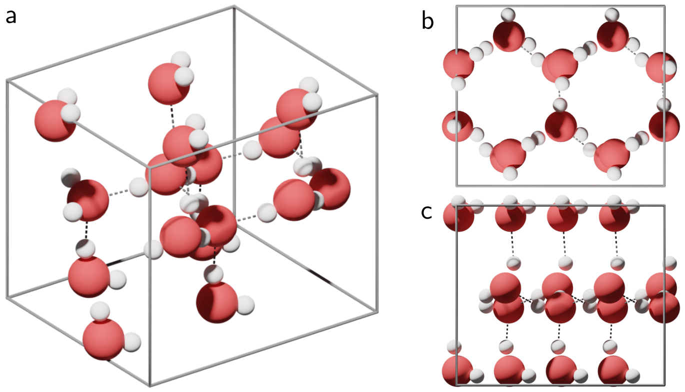


**Supplementary Figure 6**: **The crystal structure of ice XI.** (a) A $2 \times 1 \times 1$ supercell of the eight-molecule orthorhombic cell. The smaller insets show the view (b) from above and (c) from the side.

where the variational and canonical orbitals are related via a unitary rotation (i.e. $|\psi_i\rangle = \sum_j U_{ij}|\varphi_j\rangle$), and for occupied orbitals the shift simplifies to

$$\Delta\varepsilon_{i\in\text{occ}} = \sum_j \alpha_j U_{ij} U^\dagger_{ji} \left( -E_{\text{Hxc}}[\rho - n_j] + E_{\text{Hxc}}[\rho] - \int d\mathbf{r}\, v_{\text{Hxc}}[\rho](\mathbf{r}) n_j(\mathbf{r}) \right) \tag{4}$$

For a concrete example, calculations were performed on a single snapshot of liquid water with the screening parameters of all orbitals increased from 0 (i.e. the DFT solution) to 1 (the fully unscreened limit). The resulting change in the quasiparticle energies of the system are shown in Supplementary Figure 5. Note that in calculations where the screening parameter is calculated *ab initio*, each variational orbital has its own screening parameter in the range of 0.43 to 0.53. The eigenvalues change by approximately 1 eV across this window.

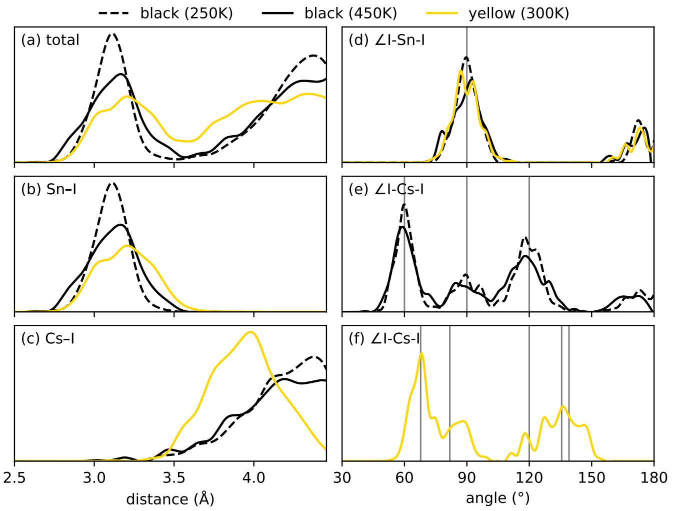


**Supplementary Figure 7**: **Comparison of coordination environments of $CsSnI_3$ for the three systems studied.** (a-c) show radial distribution functions; (d-f) show bond angle distributions. The grey vertical lines show the bond angles for pristine octahedral (six-fold), cuboctahedral (twelve-fold), and tricapped trigonal prismatic (nine-fold) coordination.

## Supplementary References

[1] Himanen, L. *et al.* DScribe: Library of descriptors for machine learning in materials science. *Comput. Phys. Commun.* **247**, 106949 (2020).

[2] Löwdin, P.-O. On the non-orthogonality problem connected with the use of atomic wave functions in the theory of molecules and crystals. *J. Chem. Phys.* **18**, 365–375 (1950).
